# The Final Frontier – Crossing the Blood-brain Barrier

**DOI:** 10.1002/emmm.201302668

**Published:** 2013-05-07

**Authors:** William S Sly, Carole Vogler

**Affiliations:** 1 Department of Biochemistry and Molecular Biology, Saint Louis University School of MedicineSt. Louis, MO, USA; 2 Department of Pathology, Saint Louis University School of MedicineSt. Louis, MO, USA

**Keywords:** gene therapy, lysosomal storage diseases, mucopolysaccharidosis, sulfamidase, transcytosis

Despite the encouraging results with enzyme replacement therapy for several lysosomal storage diseases (LSDs), successful delivery of enzyme to brain to correct central nervous system (CNS) storage has been an elusive target (Grubb et al, 2010). In that regard, the paper by Sorrentino et al (2013) in this issue is a major breakthrough. It is likely to generate widespread interest and have a large impact on research in the field.

Why is this work so important? Most of the LSDs have some element of CNS involvement. In many of them, like Sanfilippo disease type IIIA, CNS involvement is the dominant feature (Rozaklis et al, 2011). Correction of this aspect of LSDs that profoundly affects brain and leads to progressive neurological deterioration has posed the greatest challenge. Successfully meeting that challenge is a big deal!

Why is this work so important? Most of the LSDs have some element of CNS involvement. In many of them, like Sanfilippo disease type IIIA, CNS involvement is the dominant feature. Correction of this aspect of LSDs that profoundly affects brain and leads to progressive neurological deterioration has posed the greatest challenge. Successfully meeting that challenge is a big deal!

Access of corrective enzyme to lysosomes in cells of most tissues relies on receptor-mediated endocytosis, by the ubiquitously expressed mannose-6-phosphate (Man-6-P) receptor and mannose receptors on cells of the macrophage lineage. Cells in the CNS have limited access to enzymes targeting these receptors. Indeed, the blood–brain barrier (BBB) effectively blocks access of such proteins except in the perinatal period. In the mouse, access to Man-6-P receptors on the brain capillaries is limited to the first 2 weeks of life, after which the brain becomes very resistant to infused native enzyme (Urayama et al, 2008). For this reason, effective clearance of established CNS storage by infused enzymes has been quite limited.

More invasive approaches involving direct injection of enzyme into the cerebrospinal fluid intrathecally, or into the brain itself, have shown promise (Fraldi et al, 2007). In fact, clinical trials are underway to evaluate intrathecal therapy. In urgent situations like spinal cord compression, such aggressive measures are acceptable. However, more widespread application in humans may be impractical.

Some success in delivering enzyme to brain with conventional enzyme therapy, but in higher than conventional doses, has been obtained in several animal models (Vogler et al, 2005). In most cases, the correction was limited. These studies suggested that prolonged exposure to circulating enzyme, whether achieved by repeatedly infusing large doses, or by chemically modifying the enzyme to delay its clearance, enhanced the likelihood of neuronal correction. What appeared to be a significant advance in extending the circulating lifetime of β-glucuronidase by chemical modification and enhancing CNS correction in the adult mouse model of mucopolysaccharidosis (MPS) VII (Grubb et al, 2010) proved not to be useful in the murine models of MPS IIIA (Rozaklis et al, 2011) and juvenile neuronal ceroid lipofuscinosis (Batten disease) (Meng et al, 2012). These diseases and many others like them still need a breakthrough.

Sorrentino et al (2013) improved on their own prior work and that of others using several clever strategies. They chose the well-characterized murine model for MPS IIIA (Sanfilippo type IIIA). This naturally occurring mouse model has progressive neurological disease due to deficiency of sulphamidase (SGSH) which results in inability to degrade heparan sulphate in the CNS. The pathophysiology and predictable course had been very well defined by Rozaklis et al (2011), who showed that the CNS storage was resistant to even large doses of infused native or chemically modified SGSH.

Sorrentino et al (2013) reasoned they might deliver enzyme by transcytosis, targeting a receptor that delivers an essential nutrient across the BBB. Transcytosis involves endocytosis at one cell surface (*e.g.* apical) followed rapidly by exocytosis at the opposite cell surface (*e.g.* basolateral) without delivery to lysosomes. Candidate receptors include the transferrin receptor, the insulin-like growth factor receptor, and the LDL receptor (LDLR), each of which has been targeted in other work to deliver chimeric proteins to brain. Usually an antibody to the relevant receptor was used as a ‘Trojan horse’ to carry the desired protein across brain capillary endothelial cells (Zhou et al, 2012).

Spencer and Verma (2007) had successfully targeted the LDLR to deliver a virally expressed lysosomal enzyme to brain, providing proof of principle. Although correction of storage was not studied, they subsequently used this approach to deliver the protease neprilysin to brain in a transgenic Alzheimer mouse model. They showed not only efficacy in reducing brain accumulation of amyloid-beta peptide, but also improvement in CNS function (Spencer et al, 2008).

Following a similar strategy, Sorrentino et al (2013) produced a chimeric SGSH enzyme with a C-terminal extension comprising the LDLR binding domain from apolipoprotein B. As a novel twist, they substituted the signal sequence from another acid hydrolase, iduronate sulphatase (IDS), which they had found to be secreted at an unusually high rate by transfected cells. Finally, they used an adeno-associated virus vector (AAV2/8) which preferentially targets liver and incorporated the thyroxine-binding globulin promoter to achieve persistent high-level expression in liver. The goal was to make the liver a factory to secrete a continuous supply of therapeutic enzyme.

Remarkably, everything appears to have worked as predicted. They demonstrated that the AAV2/8 vector targeted the liver and that high-level expression was seen for over seven months. The predicted enhancement of secretion conferred by the IDS signal sequence was confirmed

They demonstrated that the AAV2/8 vector targeted the liver and that high-level expression was seen for over seven months. The predicted enhancement of secretion conferred by the IDS signal sequence was confirmed.

(serum levels seven- to ninefold higher). The secreted enzyme was shown to be targeted to lysosomes of mouse embryo fibroblasts *in vitro* not only by the Man-6-P receptor, but also by an endocytosis receptor which appeared to recognize the apo B domain in the chimeric enzyme, as predicted. Delivery to brain and uptake by both neurons and astrocytes was demonstrated convincingly. In addition, neuropathology and glycosaminoglycan (GAG) storage in brain were corrected.

Delivery to brain and uptake by both neurons and astrocytes was demonstrated convincingly. In addition, neuropathology and glycosaminoglycan (GAG) storage in brain were corrected.

Biomarkers for increased inflammation and aberrant autophagy that typically accompany GAG storage in brain were normalized. Finally, behavioural abnormalities were also corrected. The females, which typically become hyperactive in this model, calmed down; the males, which typically become sluggish, perked up with treatment. The appropriate controls showed that the apo B tag was required for these therapeutic responses. AAV2/8 expressing the enzyme with or without the IDS signal sequence but without the apo B tag was not effective.

All in all, it's quite a complete and remarkable story. Still, some questions remain. Are we certain that the apo B targets receptors on the BBB and promotes transcytosis across the BBB? Although this seems a plausible interpretation, could it be targeting some cells in the macrophage lineage that are recruited to brain and deliver enzyme by this route? Such cell-mediated delivery is thought to explain the CNS correction following haematopoietic stem cell replacement (Biffi et al, 2006). Is sustained high-level expression required, or could this exciting success be translated to conventional enzyme replacement therapy, where weekly or biweekly infusions are typical and brain capillaries are exposed to high levels only briefly?

Whatever the mechanism of this extraordinary CNS correction, it is likely that many laboratories will try to replicate these studies with AAV vectors expressing enzymes missing in other lysosomal diseases affecting brain (Krabbe disease, Batten disease, Tay-Sachs disease and many others). In all of these, delivery of enzymes across the BBB to correct CNS pathology and prevent the devastating cognitive impairment is the major unmet need.
